# Becoming a parent: A systematic review and meta‐analysis of changes in BMI, diet, and physical activity

**DOI:** 10.1111/obr.12959

**Published:** 2020-01-19

**Authors:** Kirsten Corder, Eleanor M. Winpenny, Campbell Foubister, Justin M. Guagliano, Xenia M. Hartwig, Rebecca Love, Chloe Clifford Astbury, Esther M.F. van Sluijs

**Affiliations:** ^1^ UKCRC Centre for Diet and Activity Research (CEDAR), MRC Epidemiology Unit, Institute of Metabolic Science University of Cambridge School of Clinical Medicine Cambridge UK

**Keywords:** emerging adulthood, mother, overweight, weight gain

## Abstract

Obesity prevalence rises fastest during young adulthood when weight, diet, and physical activity may be influenced by life events, including becoming a parent, but the impact is uncertain. We searched six electronic databases to July 2019 for longitudinal studies (both sexes) aged 15 to 35 years with a prospective pre‐pregnancy/parenthood and post‐delivery outcome. Of 11 studies (across 15 papers), six studies (women only) were eligible for meta‐analysis of the difference in change in body mass index (BMI; kg/m^2^) between remaining without children and becoming a parent. Mean (±SD) BMI gain for non‐mothers was 2.8 ± 1.3 kg/m^2^ (~7.5 kg for 164‐cm woman) over 5.6 ± 3.1 years; 12.3% of baseline BMI (22.8 ± 2.5 kg/m^2^). Becoming a mother was associated with an additional BMI increase of 0.47 ± 0.26 kg/m^2^ (~1.3 kg), 4.3% of baseline BMI (22.8 ± 5.6 kg/m^2^); the one study including men reported no difference in change. Physical activity results were equivocal; 2/4 studies (women) and 2/2 (men) showed a greater decline in parents versus non‐parents; diet (three studies) varied by dietary measure, mostly indicating no difference. Becoming a mother is associated with 17% greater absolute BMI gain than remaining childless. Motherhood BMI gain is additional to an alarming BMI increase among young women, highlighting the need for obesity prevention among all young women, including mothers.

## 1 INTRODUCTION

Inadequate physical activity and poor diet raise the risk of obesity and related metabolic disorders such as cardiovascular disease and type 2 diabetes.^1^, ^2^ Improvements in physical activity and diet could reduce obesity and cardiovascular disease burden with lifestyle accounting for up to 40% of premature cardiovascular disease related deaths.^3^ In contemporary populations, the prevalence of overweight and obesity rises faster during young adulthood (operationalized here^4^ as 15‐35 y) than during any other age,^5^ particularly among women.^6^ Young adulthood is a time characterized by a high frequency of life events, and these events have been suggested as important opportunities for health promotion as habits may be disrupted by these changes.^7^

Pregnancy itself is known to trigger deterioration in health behaviours and weight gain.^8^, ^9^, ^10^ However, pregnancy is comparatively short term, and few studies examine the long‐term effects of becoming a parent on BMI and related health behaviours for men and women (eg, diet and physical activity),^11^, ^12^, ^13^ while accounting for the background trend in the population.^14^, ^15^, ^16^ Changes in health and behaviour during pregnancy and immediately post‐partum may interact in a “vicious cycle” to increase the chances of subsequent poor behaviour and health.^16^, ^17^

For both men and women, becoming a parent is marked by major changes in social roles which co‐occur with physiological (eg, inflammatory function), psychological (eg, stress), and behavioural changes (eg, lack of sleep).^18^, ^19^, ^20^ While the onset of parenthood is often accompanied by joy, it is tempered with heightened stress and potentially harmful changes in behaviour including poor diet, low physical activity, and sleep deprivation.^20^, ^21^, ^22^, ^23^ These major changes mean that becoming a parent can have a long‐lasting impact in shaping health trajectories into midlife.^24^ For both mothers and fathers, the transition to parenthood has been suggested as a turning point for obesity with weight gains maintained long term.^12^, ^14^ Relatively few studies have examined the impact of parenthood on behaviours beyond sleep,^20^, ^21^ with limited evidence summarizing change in physical activity and diet over the transition to parenthood.^25^

Due to population level weight gain in young adulthood,^5^ and diet and physical activity being the main behavioural antecedents of obesity, we aimed to systematically review the published longitudinal observational literature to meta‐analyse evidence describing how BMI, physical activity, and diet change during young adulthood when becoming a parent compared with remaining without children (for both men and women). We incorporated a broad definition of young adulthood (15 to 35 y) as it represents a critical window for adult health, with marked changes in health and behaviour linked to developing social roles, which may also provide specific opportunities for intervention.^20^

## 2 METHODS

This review was part of a suite of reviews examining the impact of a range of life events (including leaving school, starting work, entering further education, marriage and cohabitation, as well as parenthood) on BMI, diet, and physical activity over young adulthood. We conducted a systematic literature search of longitudinal observational studies providing (non‐intervention) data on BMI, diet, and/or physical activity in men and women aged between 15 and 35 years old assessed over the transition to becoming a parent and compared with participants not becoming a parent where possible (PROSPERO ref: CRD42018106943). We searched six electronic databases (MEDLINE, Embase, PsycINFO, Scopus, ASSIA, and Web of Science) until July 2018. The search was updated in July 2019; the updated information was combined with that in the original search for clarity. The search strategy focused on three themes: outcomes (including BMI, physical activity, and diet), study type (including longitudinal and prospective), and transitions (including pregnancy and parenthood). The full list of terms is available in Table S1. The search was undertaken in parallel with a search considering additional young adulthood transitions (eg, entering work/further education and cohabitation), so the search strategy also contains terms relevant to other transitions.

### 2.1 Inclusion criteria

Inclusion criteria are presented in Table 1. Inclusion was restricted to published longitudinal data with at least two data collection points within the specified age range (15‐35 y); at least one measurement was required to be pre‐parenthood and to include prospective measurement of an outcome (ie, not only a measurement or recall during pregnancy) and at least one measurement after becoming a parent (Table 1). Retrospectively reported values were not included (eg, pre‐pregnancy values recalled post‐partum). Papers reporting measured or self‐reported BMI or body weight were included. Any measurement of physical activity or sports participation was included (self‐reported or objectively measured) as were any measures of dietary intake or eating behaviour (but not studies reporting only alcohol intake). We refer to parenthood throughout so as to include families having children without pregnancy in the household (eg, surrogacy and adoption). We restricted this review to comparing those having their first child and those remaining childless and did not examine the impact of having subsequent children. The smoking literature suggests that behaviour change is most likely to occur with the first child and, if it does not happen then, is less likely to occur with further children.^26^ Data for both men and women were eligible for inclusion. We included adolescents as young as 15 years to capture transitions occurring from mid‐adolescence to early‐adulthood and limited the age range to 35 years, which has been suggested as the end of young adulthood with regard to weight control.^4^ Studies that did not report an outcome over at least two time points were excluded (including some tracking studies or those only reporting a percentage of participants meeting guidelines). All articles published in the English language in a scientific journal, regardless of country of origin, were considered for inclusion. The search covered adiposity broadly but all studies provided, or allowed, an estimate of BMI, so this was presented; no other measures were available in enough studies to warrant inclusion.

**Table 1:** Inclusion criteria

	Inclusion Criteria	Exclusion Criteria
Setting	All countries	None
Participants	Those aged between 15 and 35 y, inclusive (at least 2 time points within that range)	Those aged below 15 y of age or above 35 y of age
		Participant groups selected based on a pre‐existing health condition (including pre‐diabetes but excluding weight status)
Exposure	Life transition, having children, parenthood, becoming a parent	Papers that do not include analysis pre and post becoming a parent
Outcomes	Individual level change:	Studies including no dietary, PA, health outcomes
	Diet: intake of energy, macronutrients, food groups, dietary patterns	Studies reporting tracking of outcomes only with no data on absolute change in behaviour
		Studies reporting solely on alcohol intake
		Studies reporting on eating disorders or weight reduction behaviour
		Studies reporting on dietary supplements
	Eating behaviours: eating outside the home, take away consumption, eating with family or friends, meal and snack consumption, cooking.	
	Physical activity: moderate‐to‐vigorous physical activity, vigorous physical activity, light physical activity, total activity, Sport participation, active travel, Energy expenditure.	
	Weight‐related outcomes	
	Adiposity‐related outcomes	
	Meta‐analysis only: Non‐parents and parents becoming a parent during course of study, with data reported separately for both participant groups	
Study type	Longitudinal prospective quantitative studies, with data reported including on specified outcomes before and after becoming a parent	Other quantitative study types
		Qualitative study
	Observational analyses of longitudinal data that were originally collected as part of an intervention/trial but reported as a separate analysis	
		Intervention studies/trial analyses
		Reviews
		Case‐control studies
		Retrospective
Publication type	Journal article	Conference abstract, study protocol, report, dissertation, book and professional journal
Publication year	Any	
Language	English	All other languages

Due to the scope and size of the overall review project, five reviewers conducted title/abstract screening. To ensure consistency in the review process, two sets of three reviewers independently reviewed three sets of 500 titles/abstracts. Iterative comparison and discussion allowed for development of a consistent screening approach; screening of all remaining titles/abstracts was then divided equally. Subsequently, two reviewers independently screened all full texts for inclusion and discussed discrepancies to reach consensus. Reference searching of the articles included identified three additional papers; none of which were included after full text screening.

### 2.2 Data extraction

One reviewer conducted data extraction with 100% checked for accuracy; discrepancies were resolved by discussion. Data were extracted separately for individuals becoming parents and those who remained childless (if available). Data extracted included baseline date (date at first point of data collection), study name, country, ethnicity, sex, socio‐economic status; and number of participants, age, outcome and measurement method, and outcome data at each relevant time point. We also recorded whether analysis of difference in change between those becoming parents and those remaining without children was conducted, including whether covariates were included in the models. Where data for men and women were included, data were extracted for males and females separately. Most of the papers only included women, and if so, data for the whole sample were extracted where possible; data for other subgroups were extracted if an overall measure by sex was not available.^27^, ^28^, ^29^ Where data from the same study were reported in multiple papers, papers with a non‐parent comparison group and also those including data for both men and women were prioritized.

### 2.3 Assessment of risk of bias

Risk of bias was assessed using an adapted version of the Effective Public Health Practice Project Quality Assessment Tool, which has been used in other physical activity reviews.^30^, ^31^ It rates participant representativeness, study size, participant drop‐out, quality of outcome data, and quality of change analyses. These criteria were assessed by two independent reviewers for all included studies; in case of disagreement, consensus was derived by discussion with a further reviewer. Each item was rated as “strong,” “moderate” or “weak”; if a paper provided insufficient information, it scored “weak.” Scores for each item were summed, and quality was defined as “weak” when at least two items were classed as “weak.” Papers were classed as “strong” when three out of the five criteria were rated as “strong,” and no items were scored as “weak”; other studies were classed as “moderate.” Further details of the instrument and scoring are provided in Table S2.

### 2.4 Data preparation

Due to the data available, wherever possible, values from all included studies including weight and height measures were converted to BMI (kg/m^2^). Minimal data conversion was necessary for meta‐analysis; for one study, mean reported weight was converted to BMI using mean height,^28^ and for another, as a measure of variation of change was unavailable, this was based on another study with a similar effect size.^32^

Data were stratified for individuals who did and did not become first‐time parents and change calculated between time points. If multiple time points were available, the data from the widest time interval (but within our exclusion criteria) were included; this decision was due to our focus on the long‐term impact of parenthood. Sample size at follow‐up(s) was assumed to be that of baseline when time‐point–specific participant numbers were not provided. Where reported, medians, inter‐quartile ranges, and variance were converted to mean and standard deviation.^33^

### 2.5 Statistical analysis

Data for the difference in change in BMI between parents and non‐parents were combined in Stata using a random effects meta‐analysis (Stata version 15, StataCorp, USA). A fixed‐effects model was run for comparison (Figure S1). Given that random effect models can overestimate intervention effect sizes in comparison with fixed‐effect models, a comparison of models can reveal the presence of small study effects that may result from publication or other biases. As both fixed (Mantel Haenszel) and random effects (DerSimonian and Laird) models produced comparable results, the analysis is presented using random effects estimates. As all studies included in the meta‐analysis were converted to a common metric, non‐standardized weighted mean differences were calculated. Variation attributable to heterogeneity was assessed using the *I* ^2^ statistic. If heterogeneity (*I* ^2^ > 50%) was seen,^34^ meta‐regression was used to test the impact of potential effect modifiers (mean baseline age, mean baseline BMI, mean follow‐up time, ethnicity [non‐White vs White], and socio‐economic status [high education vs low]). Effect modifiers were selected based on the data extracted and those used in previous reviews.^31^, ^34^ Risk of bias score was not investigated as a potential effect modifier due to limited variability. Age and follow‐up time were calculated using mean ages reported (and/or time between measurements where relevant). Variables that were associated (*P* < .05) in single models were included in a multiple model to explore the variance that could be explained. Funnel plot asymmetry and Eggers test for bias were conducted for the meta‐analyses to investigate potential publication bias. Only one study included data for men^35^ (the rest only reported on data for women), so only data for women were included in the meta‐analysis. Furthermore, three studies did not include a comparison group who did not become parents,^36^, ^37^, ^38^ and there were limited studies including physical activity and diet data; these results are presented narratively.

The results are reported in accordance with relevant guidance (Meta‐Analysis of Observational Studies in Epidemiology **[**MOOSE**]** 39 and **p**referred reporting items for systematic reviews and meta‐analyses: the PRISMA statement **[**PRISMA**]** 40).

## 3 RESULTS

**Figure 1:**
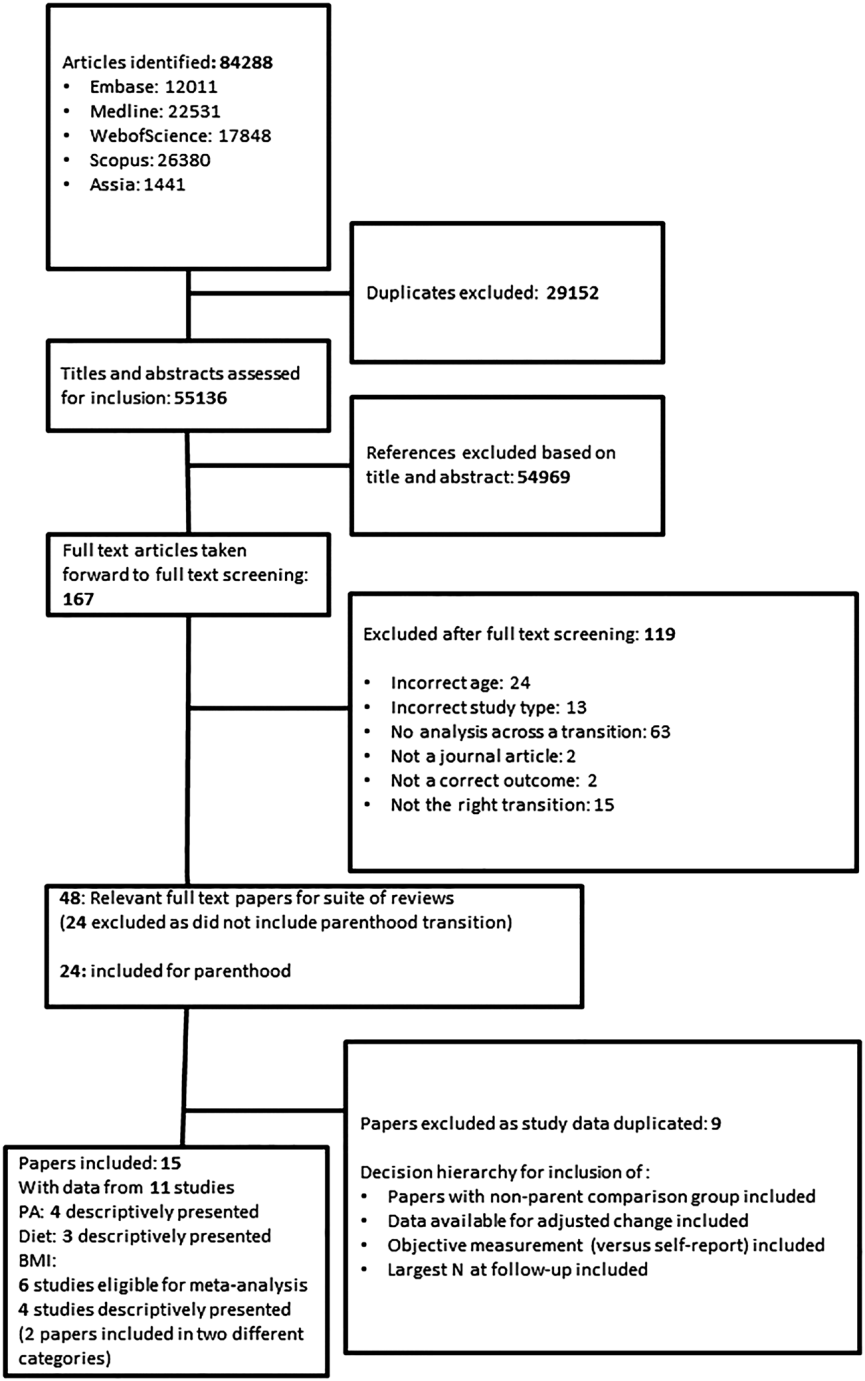
Evidence search and exclusion process

This review was part of a suite of reviews examining the impact of a range of life events (including leaving school, starting work, entering further education, marriage and cohabitation, as well as parenthood) on BMI, diet, and physical activity over young adulthood. For the full review project, after duplicate records had been excluded, the remaining 55 136 papers had titles and abstracts assessed for inclusion; 54 969 papers were excluded based on title/abstract. Of the remaining 167 papers taken forward to full text screening, 119 were excluded with the reasons for exclusion shown in Figure 1. The 48 full text papers remaining were split by life event with 24 taken forward as relevant to parenthood. Of these, nine full text papers were excluded as they included duplicate data available in other included papers. This left 15 papers from 11 studies; data from six of these were eligible for meta‐analysis. Table 2 summarizes the descriptive characteristics of the included studies; detailed study characteristics for all included studies are available in Table S3.

**Table 2:** Descriptive characteristics of the included studies, presented by outcome and as those that were meta‐analysed and those that were descriptively summarized (n = 15 papers, n = 2 included twice, from 10 studies)

	Meta‐analysed BMI	Non‐meta‐analysed BMI	Diet	Physical Activity
	N reporting (n/6)	N reporting (n/4)	N reporting (n/3)	N reporting (n/4)
Sample size (n)	6	4	3	4
<100	0	2	0	0
100‐499	0	0	0	0
500‐999	1	1	1	1
>1000	5	1	2	3
Age at baseline	22.8 (5.1)	25.3 (6.6)	28.0 (3.3)	22.4 (2.9)
Mean (SD) years				
Length of follow‐up	5.6 (3.1)	2.1 (3.3)	6.0 (1.0)	5.5 (4.4)
Mean (SD) years				
Region (n)	6	4	3	4
Australia	1	0	2	1
North America	4	4	1	3
Europe	1	0	0	0
Ethnicity (n)	(5)	(3)	(1)	(3)
% White mean (SD)	54.5 (34.7)^a^^,^ b	85.4 (15.4)	61.1 (N/A)	64.2 (19.3)^b^
SES^c^ (n)	(4)	(3)	(2)	(2)
% mothers with college degree mean (SD)	32.8 (23.0)	59.0 (25.2)	47.6 (1.5)	34.3 (25.0)

a Born in Australia classed as White ethnicity.

b Assumes that population group defined as non‐Black is of White ethnicity.

c Socio‐economic status (SES) expressed as a percentage with a college degree (the most commonly reported measure).

Table S4 presents the results of individual item and overall risk of bias assessment scores. Agreement of 100% was achieved between the two assessors for the 15 included studies. Of the 15 papers included, 12 were scored as “weak.”

### 3.1 Change in BMI

**Figure 2:**
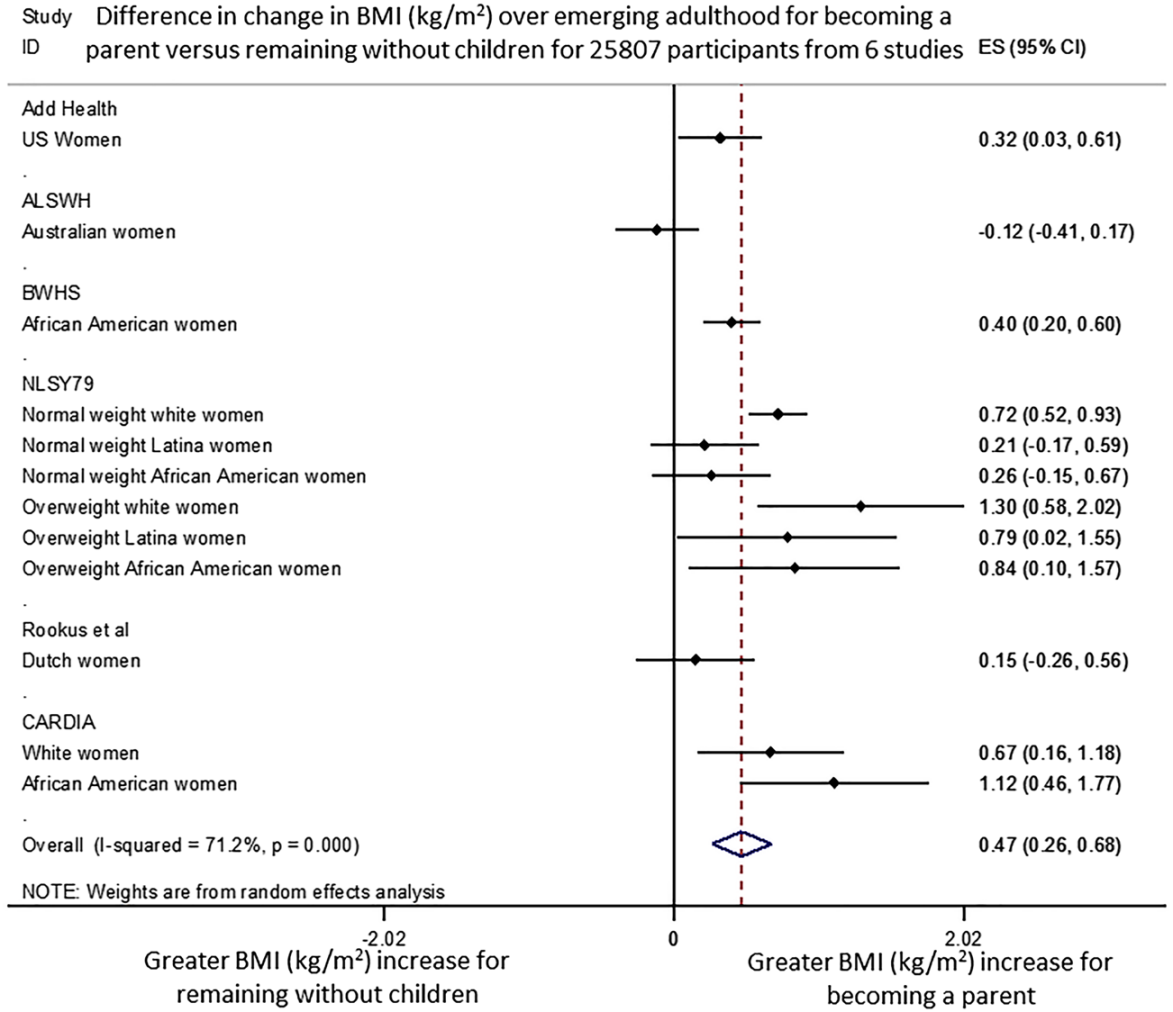
Difference in change in BMI (kg/m^2^) between women becoming mothers and those remaining without children from all eligible studies [Colour figure can be viewed at http://wileyonlinelibrary.com]

Figure 2 shows the pooled weighted mean differences, indicating that mean (±SD) BMI gain for young adult women remaining without children was 2.8 ± 1.3 kg/m^2^. Becoming a parent was associated with an additional mean BMI increase, 0.47 ± 0.26 kg/m^2^. These changes occurred over an average follow‐up of 5.6 ± 3.1 years. Therefore, the mean annual increase of BMI was 0.5 ± 0.2 kg/m^2^, with an additional increase of 0.08 ± 0.05 kg/m^2^ for those becoming parents. Compared with baseline BMI values of 22.8 ± 5.6 kg/m^2^ for parents and 22.8 ± 2.5 kg/m^2^ for non‐parents, these differences are equivalent to 14.3% and 12.3% of baseline BMI, respectively. The increase in BMI is 17% greater for parents relative to non‐parents. Heterogeneity was high (*I* ^2^ = 71.2%), and no predictors explained this variation (baseline age, ethnicity, education, baseline BMI, time of follow‐up; all *P* > .05). Funnel plot asymmetry (Figure S2) and Eggers test for bias suggested no evidence of asymmetry in the meta‐analysis (*P* = .83), indicating no evidence of publication bias.

All four studies that included BMI but were ineligible for meta‐analysis^35^, ^36^, ^37^, ^38^ presented data for women and showed either an adjusted BMI increase over time (0.32 ± 0.15 kg/m^2^)^35^ or unadjusted increases in weight gain (+2.0 kg [SD for change unavailable],^36^ +15.2 ± 2.4 kg^37^ and +4 kg [SD for change unavailable]^38^) when becoming a parent; however, not all were compared with non‐parents.^37^, ^38^ The one study including data on men reported no difference in BMI change (−0.07 ± 0.2 kg/m^2^) compared with non‐parents.^35^

### 3.2 Diet

The summary data of change in diet for becoming a parent are presented in Table 3. Results across studies could generally not be combined because of the different assessment methods and diet variables examined. Two studies used self‐reported food frequency questionnaires,^29^, ^42^ and one used an interviewer administered diet history questionnaire.^41^ Of the three studies including diet,^29^, ^41^, ^42^ two tested the difference in diet change between becoming a parent and remaining childless^41^, ^42^ and the other did not directly compare change between parents and non‐parents.^29^ A variety of dietary outcomes were reported from two Australian studies,^29^, ^42^ and one study from the United States.^41^ For both men and women, no difference in change in Dietary Guideline Index score (range 0 to 150) was found between parents and non‐parents in an Australian cohort (women: −0.5 ± 43.2; men: −3.43 ± 44.0).^42^ The US study found the percentage of energy from fat increased more among non‐parents than those becoming parents (−1.59% for parents vs −2.1% for non‐parents; *P* = .001 comparing adjusted means).^41^ Energy intake, fruit intake, fast food, and sugar sweetened beverage consumption did not differ between parents and non‐parents^41^; however, despite inclusion of data for both men and women, results were not presented separately by sex. Lastly, Elstgeest et al found women becoming a mother (living with a partner and starting a family) and not becoming a mother (remaining with a partner and not starting a family) exhibited similar change in diet over 6 years from mean (±SD)^29^ age 27.6 ± 1.5 years. Both groups of women significantly increased their fruit intake (+0.159 ± 0.019 g/d for becoming a parent vs +0.125 ± 0.032 g/d for not becoming a parent), neither changed meat consumption (+0.012 ± 0.02 g/d for becoming a parent vs +0.014 ± 0.033 g/day for not becoming a parent), but only women becoming mothers increased their cooked vegetable consumption (+0.126 ± 0.02 g/day vs +0.053 ± 0.033 g/d).^29^ Women becoming mothers increased their high fat/sugar score (+0.150 ± 0.021 g/d), whereas the score reduced among women remaining without children (−0.201 ± 0.035 g/d).

**Table 3:** Summary of diet and physical activity results

Reference	Study	Dietary Measure	Becoming a Parent	Remaining Childless
Eltgeest et al^29^, ^a^	ALSWH	Food frequency questionnaire		
		Energy intake	↑	↓
		Mediterranean diet score	o	↑
		High fat/sugar score	↑	↓
		Meat intake	o	o
		Fruit intake	↑	↑
		Cooked vegetables	↑	o
Laroche et al^41^, ^b^	CARDIA	Interviewer‐administered diet history		
		Energy intake	o	
		% energy from fat	↓↓	
		Fruit intake	o	
		Fast food	o	
		Sugar sweetened beverages	o	
Smith et al^42^, ^b^	CDAH	Food frequency questionnaire		
		Dietary Guideline Index score women	o	
		Dietary Guideline Index score men	o	

		Physical activity measure	Becoming a parent versus remaining childless	
			Women	Men
Bell et al^43^, ^b^	ALSWH	Frequency of vigorous and less‐vigorous exercise^c^	↓↓	
Hull et al^44^, ^b^	PittPAS	Past Year Leisure Time PAQ hours/week MVPA	o	↓↓*
Miller et al^45^, ^b^	Project EAT	Godin Shepard Questionnaire hours/week MVPA	↓↓*	↓↓*
Smith et al^28^, ^b^	CARDIA	Self‐reported questionnaire	↓#	
		Physical activity score		

*Note*. ↓↓, Significantly greater decrease between remaining childless and becoming a parent; ↓, significant decrease within group; ↑, significant increase within group; o, no significant difference within or between groups; #, significant unadjusted between group difference for White women across three groups, nulliparas, primiparas, and multiparas, but no difference between non‐parents and becoming a parent. Abbreviations: ALSWH, Australian Longitudinal Study of Women's Health; CARDIA, Coronary Artery Risk Development in Young Adults; CDAH, Childhood Determinants of Adult Health; PittPAS, Pittsburgh Physical Activity Study; PAQ, physical activity questionnaire; MVPA, moderate to vigorous physical activity.

a Study did not test differences between those becoming a parent and remaining childless.

b Study tested adjusted difference in change between becoming a parent and remaining childless.

c Different questions used at baseline and follow‐up.

* *P* = .05 for change between remaining childless and becoming a parent.

### 3.3 Physical activity

Four papers presented physical activity across the transition to becoming a parent, with the results summarized in Table 3.^28^, ^43^, ^44^, ^45^ All four studies included self‐reported physical activity data, and all compared physical activity between adults becoming parents and those remaining without children, two studies included men.^44^, ^45^ Physical activity data were self‐reported as the number of hours of physical activity per week over the last year,^44^ a physical activity score,^28^ the odds of being inactive for mothers versus non‐mothers,^43^ and hours/week of MVPA.^45^ Among Australian women, mothers had higher odds than non‐mothers of being inactive at follow‐up (OR = 2.2; 95% confidence interval [CI], 1.9‐2.5).^43^ In a cohort of American women, the change in activity score differed across all three parity groups for White (change ±SD: no children, −57 ± 275; first child, −118 ± 236; multiple children, +47 ± 292) but not African‐American women (no children, −8 ± 249; first child, −24 ± 335; multiple children, −19 ± 243).^28^ However, the change in activity score did not differ when directly comparing women becoming parents and women of the same ethnicity without children.^28^ Another US study found no significant difference in the magnitude of physical activity decline between mothers (−2.4 ± 3.0 h/wk) and non‐mothers (−0.1 ± 6.4 h/wk); however, the magnitude of decline among mothers could be clinically relevant. In that same study, fathers experienced a greater decrease in activity than non‐fathers (fathers, −5.0 ± 6.8 h/wk; non‐fathers, −1.5 ± 8.0 h/wk).^44^ For men, having a first child was associated with greater declines in MVPA (−1.21 ± 16.0 h/wk) between 19.4 ± 1.7 years of age and 25.3 ± 1.6 years of age, but not between 25.3 ± 1.6 years of age and 31.1 ± 1.6 years of age (−0.59 ± 13.6 h/wk). Among women, having a first child was not associated with a greater MVPA decline (−0.60 ± 11.3 h/wk) between 19.4 ± 1.7 years of age and 25.3 ± 1.6 years of age but was between 25.3 ± 1.6 years of age and 31.1 ± 1.6 years of age (−1.46 ± 10.4 h/wk).^45^

## 4 DISCUSSION

During young adulthood, becoming a mother is associated with a slightly greater increase in BMI than remaining without children. Women who become a parent increased their BMI by 14.3% of their baseline value, compared with 12.3% for women remaining without children. The increase in BMI is 17% greater for parents relative to non‐parents. The greater BMI gain when becoming a mother (0.47 ± 0.26 kg/m^2^) is additional to an alarming BMI gain (2.8 ± 1.3 kg/m^2^) for young adult women who do not have children. Diet results varied by measure and tended towards no difference in dietary change by parenthood status. Results for physical activity were equivocal but with indications of greater decreases in moderate‐to‐vigorous physical activity for men and women becoming parents compared with non‐parents. Results for BMI were from meta‐analysis, but qualitative review of collective evidence was necessary for diet and physical activity due to small numbers of included studies presenting a variety of outcomes. Parenthood results in a slight increase in BMI change compared with not becoming a parent, but all adults including non‐parents are susceptible to considerable weight changes in young adulthood. More objective longitudinal health and health behaviour data over this transition would be valuable, especially among men to better target weight management during early adulthood.^46^

### 4.1 Relationship to prior knowledge

Our results indicate that BMI increases by 2.8 ± 1.3 kg/m^2^ (~7.5 kg or ~17 lbs for an average height woman of 164 cm or 5′4″) among women during young adulthood with an additional 0.47 ± 0.26 kg/m^2^ (~1.3 kg or ~3 lbs for an average height woman) increase when becoming a mother. This supports evidence suggesting that in contemporary populations, young adulthood is an important contributor to long‐term BMI gain,^5^ particularly among women.^6^ When this increase is put in the context of the health risks of increased BMI,^47^ with data suggesting that a 5‐point BMI increase is associated with a 23% increased incidence of heart disease among British women, this early adulthood BMI gain has important health implications at the population level. This BMI increase among all young adult women aligns with population level BMI gain estimates in young adulthood in the United Kingdom.^5^ The weight gain when becoming a parent in addition to the substantial early adulthood increase in BMI is relatively small when considering that it represents a 0.08 ± 0.05 kg/m^2^ (0.4% of baseline BMI) additional annual increase, compared with 0.5 ± 0.2 kg/m^2^ per year (2.2% of baseline BMI) for early adult women who do not become parents. This review adds to previous knowledge regarding pregnancy‐related weight gain by accounting for BMI gains among non‐parents, focusing on the transition to first‐time parenthood, including men, requiring outcomes measured before pregnancy and meta‐analysing included data.^14^ Comparing motherhood BMI gain to the background trend in the population appears to have been particularly important given the large BMI gain among women not becoming mothers, which may explain the relatively small BMI increase we found for women becoming mothers, compared with 2 to 3 kg per birth stated in a previous review.^14^ However, our estimate is also slightly smaller than estimates of weight gain with parity in Australian women, stating that women with one baby gained 4 kg more than single childless women over 10 years.^48^ Previous reviews of physical activity and parenthood have compared activity levels between parents and non‐parents rather than examining change over the transition of becoming a parent.^13^, ^25^ Both previous reviews concluded that parents were less active than non‐parents^25^ and more specifically that fathers of young children spent less time in MVPA, but not sport, than childless men.^13^ Reviews examining diet during early adulthood have shown mixed longitudinal patterns, which differed by dietary outcome,^49^ with sugar consumption decreasing between late adolescence and early adulthood.^50^ However, these previous dietary reviews did not examine change in diet across life events.^49^, ^50^

We focused on life events during young adulthood as it is a critical window for adult health, where life events may amplify changes in health and behaviour linked to developing social roles.^20^ Many life events occur in young adulthood, such as leaving school, starting higher education or work, and cohabitation and/or marriage, but it is uncertain which may have the greatest impact on health and behaviour. Parenthood was shown to have a larger effect on weight gain than partnership status among Australian women,^48^ and physical activity has been shown to differ by parental status but not marriage status.^44^ It is also possible that cohabitation and parental status may interact, with non‐resident fathers experiencing different patterns of changes compared with those living with their children.^12^ A meta‐analysis of weight gain during the first year of University, traditionally termed the “Freshman 15” suggests that on average, students gain 3.38 kg (95% CI, 2.85‐3.92) over 5 months in the first year of University.^51^ In this review, the mean time of all included studies' follow‐up was 4.3 ± 3.0 years (mean 5.6 ± 3.1 y for studies included in meta‐analysis). Therefore, it appears that the BMI increase on entering higher education may be steeper than that of parenthood, but there appears to be little evidence on whether student weight gain is sustained longer term.

The inconclusive results for dietary change may be at least partly due to parental status influencing the same behaviour in different ways, for example, take away consumption may increase for some parents due to time constraints or reduce for others for economic reasons.^42^ Half of the included studies showed that women becoming a mother experienced a larger decline in physical activity when compared with non‐parents; this could be explained by limited opportunities for activity for mothers who often have the largest proportion of caring responsibilities.^52^ Parents report less moderate‐to‐vigorous activity than non‐parents,^25^, ^53^ perhaps partly due to time constraints limiting organized activities (eg, going to the gym or sports). However, parents may do more light incidental intensity activity than non‐parents,^23^ which may be harder to capture by self‐report.^54^ Due to data availability, we are unable to examine the differences between change in varying intensities of activity between parents and non‐parents. The one study examining physical activity separately by sex showed that men had a larger decline than women; this may be contributed to by men having a higher level of activity at baseline and potential regression to the mean.^44^ However, it is possible that parenthood may impact mothers and fathers differently, perhaps because mothers are often primarily responsible for their children's physical activity.^55^

### 4.2 Interpretation

Although heterogeneity was high and results should be interpreted with caution, a similar pattern of results was seen across studies. We investigated potential causes of heterogeneity using meta‐regression, but no factors were significant.

Young adulthood was defined as 15 to 35 years old due to our focus on population level life events. For clarity, we did not examine the impact of having subsequent children as previous evidence states that a much larger change in behaviour and health may occur when first becoming a parent compared to having subsequent children.^20^ We excluded outcome data assessed during pregnancy as we were interested in examining differences pre‐ and post‐parenthood, and behaviour may already have changed within the first trimester of pregnancy. Some of the included data were from the 1980s and 1990s,^28^, ^38^, ^56^ and it is possible that the social context of parenting may have changed since. Many countries may now have improved parental leave from work laws; however, in the United States, where 67% of included studies are from, there is still no mandatory paid parental leave.^20^ The mean follow‐up duration of all studies included was 4.8 years and we do not know when the children were born during that period. We therefore cannot conclude anything about the trajectory of BMI gain, for example, the additional parental BMI gain may happen gradually, only during pregnancy or directly afterwards.

Not enough data on men, diet, or physical activity were available to make conclusive suggestions, and studying population‐level estimates may mask individual variations in patterns of change and the potential influence of other life events. It is also possible that the timing of life events and their interactions may be important. For example, the effect of parenthood may differ across diverse family structures. Further work exploring the nuances of health and health behaviours over early adulthood may help unravel some of these uncertainties.

### 4.3 Strengths and limitations

To our knowledge, this is the first study to meta‐analyse change in BMI in young adulthood to compare change for those who become mothers versus those who do not. A strength of this work is that sufficient homogenous data were present for BMI to perform a meta‐analysis; however, this was not the case for diet and physical activity. We acknowledge slight deviations from our published protocol as we are focusing on one life event (parenthood); information about other life events will be published separately. We did not conduct duplicate abstract screening, which is a limitation, although 3000 articles (in two sets of 1500) were triple screened to ensure consistency among the three reviewers. Further, although data extraction was 100% checked, it was not extracted in duplicate. We did not approach authors for additional information as we did not want to bias this review in the event of differential author response.^57^ Despite a large number of papers identified in our original search, relatively few studies were included in the synthesis, which suggests a paucity of data including measures of anthropometry, physical activity, or diet before and after parenthood. This search for this review was for a suite of reviews examining the impact of a range of life events; the words pregnancy and parenthood were not included as search terms. However, as no further papers were included after manual searches of reference lists of included papers, we are confident that our search was adequate. The review aimed to characterise differences in change for individuals becoming parents versus those remaining childless; therefore, we did not use all time points separately; where possible, the first baseline time point and the latest post‐pregnancy measure within the included age range were included. The included studies were all published in English and all from North America, Australia, and Europe, so these results are not likely to be generalizable to other parts of the world. However, there was substantial diversity with regard to ethnicity with 54% of the participants included in the BMI meta‐analysis of White ethnicity. We only included peer‐reviewed publications, which may be susceptible to publication bias; however, a funnel plot did not indicate evidence of asymmetry. We chose not to include sedentary behaviour in this review for several reasons. Firstly, we wanted to focus on diet and physical activity as the main behavioural antecedents of obesity acknowledging that sedentary behaviour may primarily impact weight through diet (eg, increased snacking) or by displacing physical activity.^58^ Further, the association between objectively measured sedentary time and weight gain is relatively limited,^59^ with screen behaviours often used as proxies for sedentary time. As the screen use landscape is rapidly changing, historical data on screen use may have limited relevance to contemporary sedentary behaviour and screen time.

### 4.4 Implications for policy, practice, and research

BMI increases for women over young adulthood, with a significantly greater, but small, increase among those becoming a mother. Parenthood is potentially a “teachable moment” as new parents could be particularly willing to change their behaviour as it may also positively influence their children, rather than solely improve their own health.^60^ Parental modelling can be an important predictor of child behaviour,^61^ and recent evidence indicates that active mothers have more active 4‐ and 6‐year‐old children.^52^ Therefore aiming interventions to increase parents' activity levels may also be an additional route to increasing child activity levels. Examining messages given to new parents by health practitioners would be helpful as qualitative work has suggested widespread confusion among new mothers regarding acceptable pregnancy‐related weight gain^62^; it is also likely that this confusion may extend to diet and physical activity.

Continued efforts to prevent an increase in, and ideally reduce, BMI during young adulthood could have important public health benefits. More high‐quality longitudinal physical activity data over this time, particularly for well‐measured physical activity and diet, and among men would be valuable. This would be helpful to better characterise patterns in health and health behaviour over this time and to better inform how health promotion interventions might most effectively target this important group.

## 5 CONCLUSION

Published longitudinal data indicate that a small but significantly greater weight gain occurs when becoming a mother, in addition to an alarming BMI gain for young adult women without children. These results highlight the need for obesity prevention among young women at a population level and also confirm the importance of motherhood as a potential additional intervention target. Limited data rendered results for physical activity and diet equivocal, although moderate‐to‐vigorous physical activity may decline more among those becoming parents. More high‐quality longitudinal behavioural data among young adults becoming parents would be valuable, especially among men and on associated behaviours (eg, diet and physical activity) to better inform how to reduce BMI gain over young adulthood.

## FUNDING INFORMATION

Funding for this study and the work of all authors was supported, wholly or in part, by the Centre for Diet and Activity Research (CEDAR), a UKCRC Public Health Research Centre of Excellence (RES‐590‐28‐0002). Funding from the British Heart Foundation, Department of Health, Economic and Social Research Council, Medical Research Council, and the Wellcome Trust, under the auspices of the UK Clinical Research Collaboration, is gratefully acknowledged. The work of Kirsten Corder, Eleanor Winpenny, and Esther MF van Sluijs was supported by the Medical Research Council (MC_UU_12015/7). Rebecca Love is funded by a Gates Cambridge Scholarship. Campbell Foubister is funded by an NIHR School for Public Health PhD Studentship.

## CONFLICT OF INTEREST

The authors declare no competing interests.

## Supporting information


**Figure S1.** Difference in change in BMI (kg/m^2^) between women becoming mothers and those remaining without children from all eligible studies using fixed‐effects meta‐analysis.

**Figure S2.** Funnel plot for asymmetry showing in change in BMI (kg/m^2^) between women becoming mothers and those remaining without children and study size.

Table S1. Search terms.

## References

[1] Khaw K‐T , Wareham N , Bingham S , Welch A , Luben R , Day N . Combined impact of health behaviours and mortality in men and women: the EPIC‐Norfolk Prospective Population Study. PLoS Med. 2008;5(1):e12. 18184033 10.1371/journal.pmed.0050012 PMC2174962

[2] Benjamin EJ , Virani SS , Callaway CW , et al. Heart disease and stroke statistics‐2018 update: a report from the American Heart Association. Circulation. 2018;137(12):e67‐e492. 29386200 10.1161/CIR.0000000000000558

[3] Schroeder S . We can do better—improving the health of the American people. N Engl J Med. 2007;357:1221‐1228. 17881753 10.1056/NEJMsa073350

[4] Lytle LA , Svetkey LP , Patrick K , et al. The EARLY trials: a consortium of studies targeting weight control in young adults. Transl Behav Med. 2014;4(3):304‐313. 25264469 10.1007/s13142-014-0252-5 PMC4167899

[5] Johnson W , Li L , Kuh D , Hardy R . How has the age‐related process of overweight or obesity development changed over time? Co‐ordinated analyses of individual participant data from five United Kingdom birth cohorts. PLoS Med. 2015;12(5):e1001828. discussion e 25993005 10.1371/journal.pmed.1001828 PMC4437909

[6] Kimokoti RW , Newby PK , Gona P , et al. Patterns of weight change and progression to overweight and obesity differ in men and women: implications for research and interventions. Public Health Nutr. 2013;16(8):1463‐1475. 22939318 10.1017/S1368980012003801 PMC10271706

[7] Wethington E . An overview of the life course perspective: implications for health and nutrition. J Nutr Educ Behav. 2005;37(3):115‐120. 15904574 10.1016/s1499-4046(06)60265-0

[8] Linne Y , Dye L , Barkeling B , Rossner S . Weight development over time in parous women—the SPAWN study‐‐15 years follow‐up. Int J Obes Relat Metab Disord. 2003;27(12):1516‐1522. 14634683 10.1038/sj.ijo.0802441

[9] Rossner S , Ohlin A . Pregnancy as a risk factor for obesity: lessons from the Stockholm Pregnancy and Weight Development Study. Obes Res. 1995;3(Suppl 2):267s‐275s. 8581786 10.1002/j.1550-8528.1995.tb00473.x

[10] Luoto R , Mannisto S , Raitanen J . Ten‐year change in the association between obesity and parity: results from the National FINRISK Population Study. Gend Med. 2011;8(6):399‐406. 22153883 10.1016/j.genm.2011.11.003

[11] Saxbe D , Corner GW , Khaled M , Horton K , Wu B , Khoddam HL . The weight of fatherhood: identifying mechanisms to explain paternal perinatal weight gain. Health Psychol Rev. 2018;12(3):294‐311. 29712505 10.1080/17437199.2018.1463166

[12] Garfield CF , Duncan G , Gutina A , et al. Longitudinal study of body mass index in young males and the transition to fatherhood. Am J Mens Health. 2016;10(6):Np158‐np67. 26198724 10.1177/1557988315596224 PMC5293174

[13] Pot N , Keizer R . Physical activity and sport participation: a systematic review of the impact of fatherhood. Prev Med Rep. 2016;4:121‐127. 27413672 10.1016/j.pmedr.2016.05.018 PMC4929128

[14] Gunderson EP , Abrams B . Epidemiology of gestational weight gain and body weight changes after pregnancy. Epidemiol Rev. 2000;22(2):261‐274. 11218377 10.1093/oxfordjournals.epirev.a018038

[15] Lederman SA . The effect of pregnancy weight gain on later obesity. Obstet Gynecol. 1993;82(1):148‐155. 8515916

[16] Widen EM , Gallagher D . Body composition changes in pregnancy: measurement, predictors and outcomes. Eur J Clin Nutr. 2014;68(6):643‐652. 24667754 10.1038/ejcn.2014.40 PMC4078736

[17] Saxbe DE , Schetter CD , Guardino CM , et al. Sleep quality predicts persistence of parental postpartum depressive symptoms and transmission of depressive symptoms from mothers to fathers. Ann Behav Med. 2016;50(6):862‐875. 27492636 10.1007/s12160-016-9815-7 PMC6644068

[18] Denney JM , Nelson EL , Wadhwa PD , et al. Longitudinal modulation of immune system cytokine profile during pregnancy. Cytokine. 2011;53(2):170‐177. 21123081 10.1016/j.cyto.2010.11.005 PMC4610033

[19] Reid KM , Taylor MG . Social support, stress, and maternal postpartum depression: a comparison of supportive relationships. Soc Sci Res. 2015;54:246‐262. 26463547 10.1016/j.ssresearch.2015.08.009

[20] Saxbe D , Rossin‐Slater M , Goldenberg D . The transition to parenthood as a critical window for adult health. Am Psychol. 2018;73(9):1190‐1200. 30525801 10.1037/amp0000376

[21] Hagen EW , Mirer AG , Palta M , Peppard PE . The sleep‐time cost of parenting: sleep duration and sleepiness among employed parents in the Wisconsin Sleep Cohort Study. Am J Epidemiol. 2013;177(5):394‐401. 23378502 10.1093/aje/kws246 PMC3626047

[22] Berge JM , Larson N , Bauer KW , Neumark‐Sztainer D . Are parents of young children practicing healthy nutrition and physical activity behaviors? Pediatrics. 2011;127(5):881‐887. 21482603 10.1542/peds.2010-3218 PMC3081185

[23] Rhodes RE , Blanchard CM , Benoit C , et al. Physical activity and sedentary behavior across 12 months in cohort samples of couples without children, expecting their first child, and expecting their second child. J Behav Med. 2014;37(3):533‐542. 23606310 10.1007/s10865-013-9508-7

[24] Umberson D , Liu H , Mirowsky J , Reczek C . Parenthood and trajectories of change in body weight over the life course. Soc Sci Med. 2011;73(9):1323‐1331. 21925781 10.1016/j.socscimed.2011.08.014 PMC3391503

[25] Bellows‐Riecken KH , Rhodes RE . A birth of inactivity? A review of physical activity and parenthood. Prev Med. 2008;46(2):99‐110. 17919713 10.1016/j.ypmed.2007.08.003

[26] Tian J , Gall S , Patton G , Dwyer T , Venn A . Partnering and parenting transitions associate with changing smoking status: a cohort study in young Australians. Int J Public Health. 2017;62(8):889‐897. 28536842 10.1007/s00038-017-0984-3

[27] Abrams B , Heggeseth B , Rehkopf D , Davis E . Parity and body mass index in US women: a prospective 25‐year study. Obesity (Silver Spring). 2013;21(8):1514‐1518. 23630108 10.1002/oby.20503 PMC3752308

[28] Smith DE , Lewis CE , Caveny JL , Perkins LL , Burke GL , Bild DE . Longitudinal changes in adiposity associated with pregnancy. The CARDIA Study. Coronary Artery Risk Development in Young Adults Study. JAMA. 1994;271(22):1747‐1751. 8196117

[29] Elstgeest LE , Mishra GD , Dobson AJ . Transitions in living arrangements are associated with changes in dietary patterns in young women. J Nutr. 2012;142(8):1561‐1567. 22739375 10.3945/jn.112.158188

[30] Brown HE , Atkin AJ , Panter J , Wong G , Chinapaw MJ , van Sluijs EM . Family‐based interventions to increase physical activity in children: a systematic review, meta‐analysis and realist synthesis. Obes Rev. 2016;17(4):345‐360. 26756281 10.1111/obr.12362 PMC4819691

[31] Corder K , Winpenny E , Love R , Brown HE , White M , Sluijs EV . Change in physical activity from adolescence to early adulthood: a systematic review and meta‐analysis of longitudinal cohort studies. Br J Sports Med. 2019;53(8):496‐503. 10.1136/bjsports-2016-097330 Epub 2017 Jul 24 28739834 PMC6250429

[32] Davis D , Brown WJ , Foureur M , Nohr EA , Xu F . Long‐term weight gain and risk of overweight in parous and nulliparous women. Obesity. 2018;26(6):1072‐1077. 29687964 10.1002/oby.22174

[33] Wan X , Wang W , Liu J , Tong T . Estimating the sample mean and standard deviation from the sample size, median, range and/or interquartile range. BMC Med Res Methodol. 2014;14(1):135. 25524443 10.1186/1471-2288-14-135 PMC4383202

[34] Brooke HL , Corder K , Atkin AJ , van Sluijs EM . A systematic literature review with meta‐analyses of within‐ and between‐day differences in objectively measured physical activity in school‐aged children. Sports Med. 2014;44(10):1427‐1438. 24981243 10.1007/s40279-014-0215-5 PMC4171592

[35] Kroeger RA , Frank R . Race‐ethnicity, union status, and change in body mass index in young adulthood. J Marriage Fam. 2018;80(2):444‐462. 29773921 10.1111/jomf.12454 PMC5950716

[36] Berggren EK , Presley L , Amini SB , Hauguel‐de Mouzon S , Catalano PM . Are the metabolic changes of pregnancy reversible in the first year postpartum? Diabetologia. 2015;58(7):1561‐1568. 25957777 10.1007/s00125-015-3604-x PMC4703315

[37] Sidebottom AC , Brown JE , Jacobs DR Jr . Pregnancy‐related changes in body fat. Eur J Obstet Gynecol Reprod Biol. 2001;94(2):216‐223. 11165728 10.1016/s0301-2115(00)00329-8

[38] South‐Paul JE , Rajagopal KR , Tenholder MF . Exercise responses prior to pregnancy and in the postpartum state. Med Sci Sports Exerc. 1992;24(4):410‐414. 1560735

[39] Stroup DF , Berlin JA , Morton SC , et al. Meta‐analysis of observational studies in epidemiology: a proposal for reporting. Meta‐analysis Of Observational Studies in Epidemiology (MOOSE) group. JAMA. 2000;283(15):2008‐2012. 10789670 10.1001/jama.283.15.2008

[40] Moher D , Liberati A , Tetzlaff J , Altman DG , Group P . Preferred reporting items for systematic reviews and meta‐analyses: the PRISMA statement. J Clin Epidemiol. 2009;62(10):1006‐1012. 19631508 10.1016/j.jclinepi.2009.06.005

[41] Laroche HH , Wallace RB , Snetselaar L , Hillis SL , Steffen LM . Changes in diet behavior when adults become parents. J Acad Nutr Diet. 2012;112(6):832‐839. 22551676 10.1016/j.jand.2012.02.024 PMC3378760

[42] Smith KJ , McNaughton SA , Gall SL , Otahal P , Dwyer T , Venn AJ . Associations between partnering and parenting transitions and dietary habits in young adults. J Acad Nutr Diet. 2017;117(8):1210‐1221. 28169212 10.1016/j.jand.2016.12.008

[43] Bell S , Lee C . Emerging adulthood and patterns of physical activity among young Australian women. Int J Behav Med. 2005;12(4):227‐235. 16262541 10.1207/s15327558ijbm1204_3

[44] Hull EE , Rofey DL , Robertson RJ , Nagle EF , Otto AD , Aaron DJ . Influence of marriage and parenthood on physical activity: a 2‐year prospective analysis. J Phys Act Health. 2010;7(5):577‐583. 20864752 10.1123/jpah.7.5.577 PMC3124092

[45] Miller J , Nelson T , Barr‐Anderson DJ , Christoph MJ , Winkler M , Neumark‐Sztainer D . Life events and longitudinal effects on physical activity: adolescence to adulthood. Med Sci Sports Exerc. 2019;51(4):663‐670. 30673690 10.1249/MSS.0000000000001839 PMC7166312

[46] Deliens T , Versele V , Vanden Eynde H , et al. Body weight, body composition and energy balance related behaviour during the transition to parenthood: study protocol of a multi‐centre observational follow‐up study (TRANSPARENTS). BMC Public Health. 2019;19(1):516. 31060535 10.1186/s12889-019-6884-0 PMC6501312

[47] Canoy D , Cairns BJ , Balkwill A , et al. Body mass index and incident coronary heart disease in women: a population‐based prospective study. BMC Med. 2013;11(1):87. 23547896 10.1186/1741-7015-11-87 PMC3661394

[48] Brown WJ , Hockey R , Dobson AJ . Effects of having a baby on weight gain. Am J Prev Med. 2010;38(2):163‐170. 20117572 10.1016/j.amepre.2009.09.044

[49] Winpenny EM , Penney TL , Corder K , White M , van Sluijs EMF . Change in diet in the period from adolescence to early adulthood: a systematic scoping review of longitudinal studies. Int J Behav Nutr Phys Act. 2017;14(1):60. 28472992 10.1186/s12966-017-0518-7 PMC5418762

[50] Winpenny EM , Penney TL , Corder K , White M , van Sluijs EMF . Changes in consumption of added sugars from age 13 to 30 years: a systematic review and meta‐analysis of longitudinal studies. Obes Rev. 2017;18(11):1336‐1349. 28869998 10.1111/obr.12588 PMC5656815

[51] Vadeboncoeur C , Townsend N , Foster C . A meta‐analysis of weight gain in first year university students: is freshman 15 a myth? BMC Obes. 2015;2:22. 26217537 10.1186/s40608-015-0051-7 PMC4511069

[52] Hesketh KR , Brage S , Cooper C , et al. The association between maternal‐child physical activity levels at the transition to formal schooling: cross‐sectional and prospective data from the Southampton Women's Survey. Int J Behav Nutr Phys Act. 2019;16(1):23. 30786904 10.1186/s12966-019-0782-9 PMC6381630

[53] Carson V , Adamo K , Rhodes RE . Associations of parenthood with physical activity, sedentary behavior, and sleep. Am J Health Behav. 2018;42(3):80‐89. 10.5993/AJHB.42.3.8 29663983

[54] Corder K , van Sluijs EM , Wright A , Whincup P , Wareham NJ , Ekelund U . Is it possible to assess free‐living physical activity and energy expenditure in young people by self‐report? Am J Clin Nutr. 2009;89(3):862‐870. 19144732 10.3945/ajcn.2008.26739

[55] Solomon‐Moore E , Toumpakari Z , Sebire SJ , Thompson JL , Lawlor DA , Jago R . Roles of mothers and fathers in supporting child physical activity: a cross‐sectional mixed‐methods study. BMJ Open. 2018;8(1):e019732. 10.1136/bmjopen-2017-019732 PMC5781024 29358449

[56] Rookus MA , Rokebrand P , Burema J , Deurenberg P . The effect of pregnancy on the body mass index 9 months postpartum in 49 women. Int J Obes (Lond). 1987;11(6):609‐618. 3440682

[57] Selph S , Ginsburg A , Chou R . Impact of contacting study authors to obtain additional data for systematic reviews: diagnostic accuracy studies for hepatic fibrosis. Syst Rev. 2014;3:107. 25239493 10.1186/2046-4053-3-107 PMC4185334

[58] Must A , Tybor DJ . Physical activity and sedentary behavior: a review of longitudinal studies of weight and adiposity in youth. Int J Obes (Lond). 2005;29(Suppl 2):S84‐S96. 16385758 10.1038/sj.ijo.0803064

[59] Thorp AA , Owen N , Neuhaus M , Dunstan DW . Sedentary behaviors and subsequent health outcomes in adults a systematic review of longitudinal studies, 1996‐2011. Am J Prev Med. 2011;41(2):207‐215. 21767729 10.1016/j.amepre.2011.05.004

[60] Edvardsson K , Ivarsson A , Eurenius E , et al. Giving offspring a healthy start: parents' experiences of health promotion and lifestyle change during pregnancy and early parenthood. BMC Public Health. 2011;11(1):936. 22171644 10.1186/1471-2458-11-936 PMC3282831

[61] Campbell KJ , Crawford DA , Salmon J , Carver A , Garnett SP , Baur LA . Associations between the home food environment and obesity‐promoting eating behaviors in adolescence. Obesity (Silver Spring). 2007;15(3):719‐730. 17372323 10.1038/oby.2007.553

[62] Nikolopoulos H , Mayan M , MacIsaac J , Miller T , Bell RC . Women's perceptions of discussions about gestational weight gain with health care providers during pregnancy and postpartum: a qualitative study. BMC Pregnancy Childbirth. 2017;17(1):97. 28335749 10.1186/s12884-017-1257-0 PMC5364680

