# Supplementary figures and images for: Becoming a parent: A systematic review and meta‐analysis of changes in BMI, diet, and physical activity
Source: Obes Rev. 2020 Jan 19;21(4):e12959. doi: 10.1111/obr.12959 (PMC7078970; doi:10.1111/obr.12959)

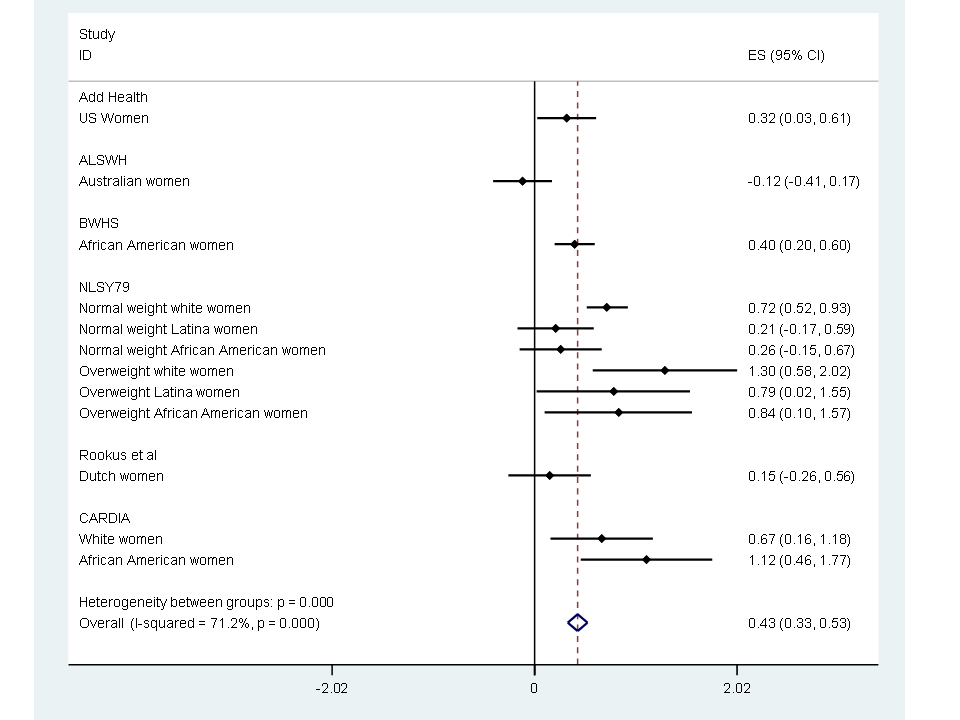

Supplement: Supplementary file 1 — Figure S1. Difference in change in BMI (kg/m2) between women becoming mothers and those remaining without children from all eligible studies using fixed‐effects meta‐analysis. [file OBR-21-e12959-s001.tif]

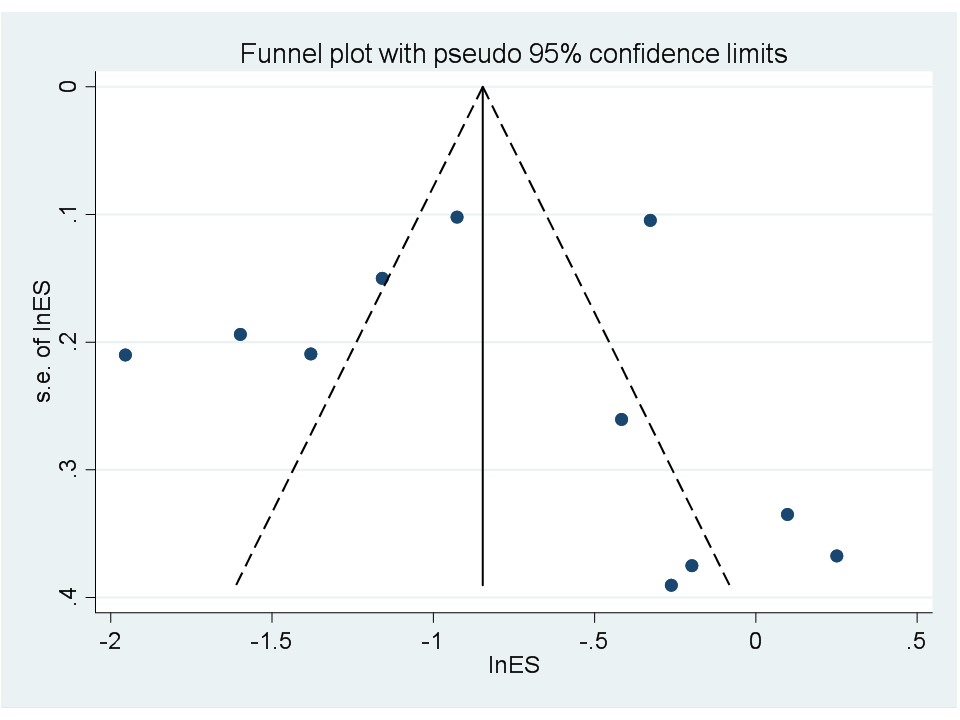

Supplement: Supplementary file 2 — Figure S2. Funnel plot for asymmetry showing in change in BMI (kg/m2) between women becoming mothers and those remaining without children and study size. [file OBR-21-e12959-s002.tif]
