# Supplementary material for: Becoming a parent: A systematic review and meta‐analysis of changes in BMI, diet, and physical activity
Source: Obes Rev. 2020 Jan 19;21(4):e12959. doi: 10.1111/obr.12959 (PMC7078970; doi:10.1111/obr.12959)
Supplement: Supplementary file 3 — Table S1. Search terms. [file OBR-21-e12959-s003.docx]

Supplementary Table 1. Search terms.

| 1 | Diet Outcomes  Physical activity outcomes  Health outcomes | food*[Title/Abstract] OR beverage*[Title/Abstract] OR nutrient*[Title/Abstract] OR macronutrient*[Title/Abstract] OR “energy intake”[Title/Abstract] OR diet[Title/Abstract] OR diets[Title/Abstract] OR “dietary”[Title/Abstract] OR nutrition[Title/Abstract] OR nutritional[Title/Abstract] OR fruit[Title/Abstract] OR vegetable[Title/Abstract] OR fruits[Title/Abstract] OR vegetables[Title/Abstract] OR dairy[Title/Abstract] OR grain*[Title/Abstract] OR meat[Title/Abstract] OR cereal*[Title/Abstract] OR “soft drink*”[Title/Abstract] OR soda[Title/Abstract] OR SSB[Title/Abstract] OR SSBs[Title/Abstract] OR salt[Title/Abstract] OR sugar*[Title/Abstract]  OR "Food"[Mesh] OR "Beverages"[Mesh] OR diet[Mesh] OR "Nutrition Surveys"[Mesh] OR "Diet Records"[Mesh] OR "Dietary Fats"[Mesh] OR "Dietary Proteins"[Mesh] OR "Dietary Carbohydrates "[Mesh] OR "Healthy Diet"[Mesh]  OR cook*[Title/Abstract] OR “food preparation”[Title/Abstract] OR “eating behaviour”[Title/Abstract] OR “eating behavior”[Title/Abstract] OR meal*[Title/Abstract] OR snack*[Title/Abstract] OR “dining out”[Title/Abstract] OR breakfast[Title/Abstract] OR dinner[Title/Abstract] OR lunch[Title/Abstract] OR supper[Title/Abstract] OR “fast food”[Title/Abstract] OR “fast-food”[Title/Abstract] OR restaurant[Title/Abstract] OR “take-away”[Title/Abstract] OR takeaway[Title/Abstract] OR “eating out”[Title/Abstract] OR “away from home”[Title/Abstract] OR “out of home”[Title/Abstract] OR “away-from-home”[Title/Abstract] OR “out-of-home”[Title/Abstract] OR “home-cook*”[Title/Abstract] OR “home prepar*”[Title/Abstract] OR “home cook*”[Title/Abstract] OR “home-prepar*”[Title/Abstract]  OR "physical activity"[Title/Abstract] OR "physical activities"[Title/Abstract] OR “physically active”[Title/Abstract] OR "active transport"[Title/Abstract] OR "active travel"[Title/Abstract] OR exercise*[Title/Abstract] OR cycle[Title/Abstract] OR cycling[Title/Abstract] OR walk*[Title/Abstract] OR sport*[Title/Abstract] OR "energy expenditure"[Title/Abstract] OR MVPA[Title/Abstract] OR “vigorous activity”[Title/Abstract] OR VPA[Title/Abstract] OR “moderate activity” [Title/Abstract] OR “light activity”[Title/Abstract] OR MPA[Title/Abstract] OR “intensity activity”[Title/Abstract] OR “LPA”[Title/Abstract] OR “strenuous activity”[Title/Abstract] OR “Exercise”[MeSH] OR “Sports”[Mesh]  OR “BMI”[Title/Abstract] OR "weight gain"[Title/Abstract] OR "weight loss"[Title/Abstract] OR "body weight"[Title/Abstract] OR "body mass"[Title/Abstract] OR "change in weight"[Title/Abstract] OR " weight change"[Title/Abstract] OR "waist circumference"[Title/Abstract] OR anthropometry[Title/Abstract] OR anthropometric[Title/Abstract] OR “fat mass”[Title/Abstract] OR “lean mass”[Title/Abstract] OR “body composition”[Title/Abstract] OR skinfold[Title/Abstract] OR overweight[Title/Abstract] OR obesity[Title/Abstract] OR adiposity[Title/Abstract] |
| --- | --- | --- |
| 2 | Longitudinal | longitudinal[Title/Abstract] OR cohort[Title/Abstract] OR prospective[Title/Abstract] OR “follow-up*”[Title/Abstract] OR "follow up"[Title/Abstract] OR "Follow-Up Studies"[Mesh] OR “Prospective Studies”[Mesh] OR “Longitudinal Studies”[Mesh] OR “Cohort Studies”[Mesh] OR “life-course”[Title/Abstract] OR “life course”[Title/Abstract] OR “repeated measure”[Title/Abstract] OR “repeated-measure”[Title/Abstract] |
| 3 | Transition | “Further education”[Title/Abstract] OR “Higher education”[Title/Abstract] OR “entering education”[Title/Abstract] OR “leaving education”[Title/Abstract] OR “leaving school”[Title/Abstract] OR “school leaver*”[Title/Abstract] OR college[Title/Abstract] OR university[Title/Abstract] OR freshman[Title/Abstract] OR freshmen[Title/Abstract] OR Graduate[Title/Abstract] OR Undergrad*[Title/Abstract] OR employment[Title/Abstract] OR “first job”[Title/Abstract] OR “starting work” [Title/Abstract] OR “moving house”[Title/Abstract] OR “change of address”[Title/Abstract] OR “residential relocation”[Title/Abstract] OR “residential mobility”[Title/Abstract] OR “residential instability”[Title/Abstract] OR “geographic* mobility”[Title/Abstract] OR “geographic relocation”[Title/Abstract] OR “residential relocation”[Title/Abstract] OR “residential stability”[Title/Abstract] OR  OR “living arrangements”[Title/Abstract] OR cohabit*[Title/Abstract] OR marriage[Title/Abstract] OR “marital status” [Title/Abstract] OR parity[Title/Abstract] OR parous[Title/Abstract] OR childbearing[Title/Abstract] OR postpartum[Title/Abstract] OR “post-partum”[Title/Abstract] OR "first child*"[Title/Abstract] OR "having children"[Title/Abstract] OR "having a child"[Title/Abstract] OR trajector*[Title/Abstract] OR “transition*”[Title/Abstract] OR “life transition”[Title/Abstract] OR “life events”[Title/Abstract] OR “Life Change Events"[Mesh] OR "Marital Status"[Mesh] OR "Employment"[Mesh] |
| 4 | Additional filters | English[lang]  NOT Review[ptyp]  NOT Neoplasm[Mesh]  Humans (filter) |
| 5 |  | 1 AND 2 AND 3 AND 4 |

Note. The search strategy was originally designed for PubMed and then adapted as necessary for the other databases.

Supplementary Table 2. Risk of bias scoring criteria, adapted from the Effective Public Health Practice Project Quality Assessment Tool.

| **Characteristic** | **Question** | **Scoring** | **Scoring** |
| --- | --- | --- | --- |
| **Representativeness** | Are the individuals selected to participate in the study likely to be representative of the target population? | 1 Very likely  2 Somewhat  3 Not likely  4 Can’t tell | Strong  Moderate  Weak  Weak |
|  | What percentage of selected individuals agreed to participate? | 1 80-100%  2 60–79%  3 <60%  4 Not applicable  5 Can’t tell | Strong  Moderate  Weak  Weak  Weak |
| **Number of participants** | How many participants were in the study? | 1 >1000  2 999-101  3 <100 | Strong  Moderate  Weak |
| **Drop-outs** | Were withdrawals and drop-outs reported in terms of numbers and/or reasons per group? | 1 Yes  2 No  3 Can’t tell  4 Not Applicable | Strong  Weak  Weak  Weak |
|  | Indicate the percentage of participants completing the study. (If the percentage differs by groups, record the lowest). | 1 80-100%  2 60–79%  3 <60%  4 Not applicable  5 Can’t tell | Strong  Weak  Weak  Weak  Weak |
| **Data collection** | Was the tool objective or subjective? | 1 Objective  2 Reported  3 Can’t tell | Strong  Weak  Weak |
|  | Was the tool valid? | 1 Yes  2 No  3 Can’t tell | Strong  Weak  Weak |
|  | Was the tool reliable? | 1 Yes  2 No  3 Can’t tell | Strong  Weak  Weak |
|  | Was the tool the same at all time-points? | 1 Yes  2 No  3 Can’t tell | Strong  Weak |
| **Analyses** | Was change in outcome statistically tested? | 1 Yes  2 No  3 Can’t tell | Strong/Moderate  Weak  Weak |
|  | Was adjustment for potential confounders included? | 1 Yes  2 No  3 Can’t tell | Strong/Moderate  Moderate/Weak  Weak |

Note. When multiple questions represent one category, the results of all category questions were combined to obtain a score and the lowest ranking for a category was taken. For example, if a self-reported measure of activity was reported to be valid and reliable and the same over both time-points, it was scored as ‘moderate’. If a self-reported measure of activity was not reported to be valid and reliable or was different over time-points it was scored as ‘Weak’.

Scores for each item were summed and the score was defined as ‘weak’ when at least two items were classed as ‘weak’. Papers were classed as ‘strong’ when three out of the five criteria were rated as ‘strong’ and no items were scored as ‘weak’; other studies were classed as ‘moderate’.

Supplementary Table 3. Descriptive characteristics of included papers.

| **Reference** | **Study name** | **Country** | **Date** | **N** | **% women** | **Ethnicity** | **SES** | **Baseline mean age (y)** | **Time to follow-up (y)** | **Assessment** | **Outcome** | **Meta-analysed** |
| --- | --- | --- | --- | --- | --- | --- | --- | --- | --- | --- | --- | --- |
| Abrams et al. (2013) [^1^](#_ENREF_1) | NLSY79 | USA | 2013 | 3943 | 100 | 64.2% white; 15.2% Hispanic; 20.5% black | 86.7% not in poverty | 20.1 (2.3) | 10 | Self-reported | BMI | Yes |
| Bell & Lee (2005) [^2^](#_ENREF_2) | ALSWH | Australia | 2005 | 8545 | 100 | - | - | 20.7 (1.48) | 4 | Self-reported | PA | No |
| Berggren et al. (2015) [^3^](#_ENREF_3) | - | USA | 2015 | 21 | 100 | 91% white | 82% at least college degree | 29 (27-36) | 1 | Measured | BMI | No |
| Davis et al. (2018) [^4^](#_ENREF_4) | ALSWH | Australia | 2018 | 8009 | 100 | 93.24% born in Australia | 14.49% university degree | 20.34 (1.5) | 7 | Self-reported | BMI | Yes |
| Elstgeest et al. (2012) ^[5](#_ENREF_5)^ | ALSWH | Australia | 2012 | 6534 | 100 | na | 48.6% university degree | 27.6 (1.5) | 6 | Self-reported | Diet | No |
| Hull et al. (2010) [^6^](#_ENREF_6) | PittPAS | USA | 2010 | 828 | 52 | 15% AA | 52% at least college degree | 24.7 (1.1) | 2 | Self-reported | PA | No |
| Kroeger & Frank (2018) [^7^](#_ENREF_7) | AddHealth | USA | 2018 | 11119 | 53.4 | 68% white | 36.5% college degree | 15.47 (0.14) | 7 | Measured | BMI | Yes |
| Laroche et al. (2012) [^8^](#_ENREF_8) | CARDIA | USA | 2012 | 5115 | 54 | 61.1% white | 14.4y education on average | 24.6 (SD missing) | 7 | Self-reported | Diet | No |
| Miller et al., (2019) [^9^](#_ENREF_9) | Project EAT* | USA | 2003 | 2516 | 55.1 | 60.6% white | 16.6% ‘high’ education | 19.4 (1.7) | 12 | Self-reported | PA | No |
| Rookus et al. (1987) [^10^](#_ENREF_10) | - | The Netherlands | 1987 | 915 | 100 | - | 14% university degree | 29 (SD missing) | 1.75 | Self-reported | BMI | Yes |
| Rosenberg et al. (2003) [^11^](#_ENREF_11) | BWHS | USA | 2003 | 11196 | 100 | 100% AA | 60.77% at least 16y education | 27 (SD missing) | 4 | Self-reported | BMI | Yes |
| Sidebottom et al. (2001) [^12^](#_ENREF_12) | Diana Project | USA | 2001 | 557 | 100 | 97.1% white | 63% college degree | 29.2 (3.14) | 0.12 | Measured | BMI | No |
| Smith et al. (1994) [^13^](#_ENREF_13) | CARDIA | USA | 1994 | 2788 | 100 | 47% white^a^ | 15.15y education on average | 25.1 (3.5) | 5 | Self-reported | BMI, PA | Yes |
| Smith et al. (2017) [^14^](#_ENREF_14) | CDAH | Australia | 2017 | 1402 | 61.1 | - | 50.2% university degree | 31.5 (2.7) | 5 | Self-reported | Diet | No |
| South-Paul et al. (1992) [^15^](#_ENREF_15) | - | USA | 1992 | 17 | 100 | - | - | 27.5 (2.2) | 0.14 | Measured | BMI | No |

Note. ^a^assumes that population group defined as non-black is of white ethnicity. Abbreviations: BMI = body mass index; PA = physical activity; SES = socio-economic Status, Abbreviations: NLSY79, National Longitudinal Survey of Youth 1979; ALSWH, Australian Longitudinal Study of Women’s Health; PittPAS, Pittsburg Physical Activity Study; Add Health, National Longitudinal Study of Adolescent to Adult Health; CARDIA, Coronary Artery Risk Development in Young Adults; BWHS, Black Women’s Health Study; CDAH, Childhood Determinants of Adult Health.*Wave 2 of Project EAT, first wave with parenthood assessed.

Supplementary Table 4. Risk of bias assessment scores.

| **Reference** | **Representativeness** | **Number of participants** | **Drop outs** | **Data collection** | **Analyses** | **Overall rating** |
| --- | --- | --- | --- | --- | --- | --- |
| Abrams et al. (2013) [^1^](#_ENREF_1) | Moderate | Strong | Moderate | Weak | Strong | Moderate |
| Bell & Lee (2005) [^2^](#_ENREF_2) | Weak | Strong | Strong | Weak | Moderate | Weak |
| Berggren et al. (2015) [^3^](#_ENREF_3) | Weak | Weak | Strong | Strong | Strong | Weak |
| Davis et al. (2018) [^4^](#_ENREF_4) | Weak | Strong | Weak | Weak | Weak | Weak |
| Elstgeest et al. (2012) ^[5](#_ENREF_5)^ | Weak | Strong | Weak | Weak | Strong | Weak |
| Hull et al. (2010) [^6^](#_ENREF_6) | Moderate | Moderate | Moderate | Weak | Strong | Moderate |
| Kroeger & Frank (2018) [^7^](#_ENREF_7) | Moderate | Strong | Moderate | Weak | Strong | Moderate |
| Laroche et al. (2012) [^8^](#_ENREF_8) | Weak | Strong | Weak | Weak | Strong | Weak |
| Miller et al. (2019) [^9^](#_ENREF_9) | Strong | Strong | Weak | Weak | Strong | Weak |
| Rookus et al. (1987) [^10^](#_ENREF_10) | Weak | Moderate | Weak | Strong | Moderate | Weak |
| Rosenberg et al. (2003) [^11^](#_ENREF_11) | Weak | Strong | Strong | Weak | Strong | Weak |
| Sidebottom et al. (2001) [^12^](#_ENREF_12) | Weak | Moderate | Weak | Strong | Weak | Weak |
| Smith et al. (1994) [^13^](#_ENREF_13) | Weak | Strong | Weak | Strong/Weak^1^ | Weak | Weak |
| Smith et al. (2017) [^14^](#_ENREF_14) | Weak | Strong | Weak | Weak | Strong | Weak |
| South-Paul et al. (1992) [^15^](#_ENREF_15) | Weak | Weak | Weak | Weak | Moderate | Weak |

Note. ^1^Strong for measurement of BMI and weak for measurement of physical activity.

Supplementary Table 5. Descriptive characteristics of included papers with n for parent groups.

| **Reference** | **Study name** | **% women** | **Outcome** | **Compared parent and non-parents** | **N** | **N becoming a parent** | **N not becoming a parent** | **Meta-analysed** |
| --- | --- | --- | --- | --- | --- | --- | --- | --- |
| Abrams et al. (2013) [^1^](#_ENREF_1) | NLSY79 | 100 | BMI | Yes | 3943 | White women n=353  Hispanic women n=115  Black women n=150 | White women n=619  Hispanic women n=176  Black women n=255 | Yes |
| Bell & Lee (2005) [^2^](#_ENREF_2) | ALSWH | 100 | PA | Yes | 8545 | n=1538 (calculated from %) | n=7007 (calculated from %) | No |
| Berggren et al. (2015) [^3^](#_ENREF_3) | - | 100 | BMI | Yes | 21 | n=11 | n=10 | No |
| Davis et al. (2018) [^4^](#_ENREF_4) | ALSWH | 100 | BMI | Yes | 8009 | n=1291 with one birth throughout study | n=2161 with no births | Yes |
| Elstgeest et al. (2012) ^[5](#_ENREF_5)^ | ALSWH | 100 | Diet | No | 6534 | n=1723 (from living with partner to becoming a parent) | n=575 (remaining with partner) | No |
| Hull et al. (2010) [^6^](#_ENREF_6) | PittPAS | 52 | PA | Yes | 828 | Women n=16  Men n=22 | Women n=211  Men n=221 | No |
| Kroeger & Frank (2018) [^7^](#_ENREF_7) | AddHealth | 53.4 | BMI | Yes | 11119 | Not reported | Not reported | Yes (for women, but not men) |
| Laroche et al. (2012) [^8^](#_ENREF_8) | CARDIA | 54 | Diet | Yes | 5115 | Not reported | Not reported | No |
| Miller et al., (2019) [^9^](#_ENREF_9) | Project EAT* | 55.1 | PA | Yes | 2516 | n=328 between 19.4 ± 1.7y and 25.3 ± 1.6y    n=456 between 25.3 ± 1.6y and 31.1 ± 1.6y | n=1371 between 19.4 ± 1.7y and 25.3 ± 1.6y  n=779 between 25.3 ± 1.6y and 31.1 ± 1.6y | No |
| Rookus et al. (1987) [^10^](#_ENREF_10) | - | 100 | BMI | Yes | 915 | n=49 | n=400 | Yes |
| Rosenberg et al. (2003) [^11^](#_ENREF_11) | BWHS | 100 | BMI | Yes | 11196 | n=598 | N=9966 | Yes |
| Sidebottom et al. (2001) [^12^](#_ENREF_12) | Diana Project | 100 | BMI | No | 557 | Not reported | Not reported | No |
| Smith et al. (1994) [^13^](#_ENREF_13) | CARDIA | 100 | BMI, PA | Yes | 2788 | Black women n=41  White women n=48 | Black women n=327  White women n=592 | Yes for BMI, but not PA |
| Smith et al. (2017) [^14^](#_ENREF_14) | CDAH | 61.1 | Diet | Yes | 1402 | Women n=458  Men n=216 | Women n=399  Men n=329 | No |
| South-Paul et al. (1992) [^15^](#_ENREF_15) | - | 100 | BMI | No | 17 | n=11 | Not available | No |

Note. The overall study N may not match to comparison groups as there may be other subgroups (e.g. people already with children).

Abbreviations: BMI = body mass index; PA = physical activity, Abbreviations: NLSY79, National Longitudinal Survey of Youth 1979; ALSWH, Australian Longitudinal Study of Women’s Health; PittPAS, Pittsburg Physical Activity Study; Add Health, National Longitudinal Study of Adolescent to Adult Health; CARDIA, Coronary Artery Risk Development in Young Adults; BWHS, Black Women’s Health Study; CDAH, Childhood Determinants of Adult Health.*Wave 2 of Project EAT, first wave with parenthood assessed.

References

1. Abrams B, Heggeseth B, Rehkopf D, Davis E. Parity and body mass index in US women: a prospective 25-year study. *Obesity (Silver Spring)* 2013; **21**(8): 1514-8.

2. Bell S, Lee C. Emerging adulthood and patterns of physical activity among young Australian women. *Int J Behav Med* 2005; **12**(4): 227-35.

3. Berggren EK, Presley L, Amini SB, Hauguel-de Mouzon S, Catalano PM. Are the metabolic changes of pregnancy reversible in the first year postpartum? *Diabetologia* 2015; **58**(7): 1561-8.

4. Davis D, Brown, W.J., Foureur, M., Nohr, E.A., Xu, F. Long-Term Weight Gain and Risk of Overweight in Parous and Nulliparous Women. *Obesity* 2018; **26**(6).

5. Elstgeest LE, Mishra GD, Dobson AJ. Transitions in living arrangements are associated with changes in dietary patterns in young women. *J Nutr* 2012; **142**(8): 1561-7.

6. Hull EE, Rofey DL, Robertson RJ, Nagle EF, Otto AD, Aaron DJ. Influence of marriage and parenthood on physical activity: a 2-year prospective analysis. *J Phys Act Health* 2010; **7**(5): 577-83.

7. Kroeger RA, Frank R. Race-Ethnicity, Union Status, and Change in Body Mass Index in Young Adulthood. *Journal of Marriage and Family* 2018; **80**(2): 444-62.

8. Laroche HH, Wallace RB, Snetselaar L, Hillis SL, Steffen LM. Changes in diet behavior when adults become parents. *J Acad Nutr Diet* 2012; **112**(6): 832-9.

9. Miller J, Nelson T, Barr-Anderson DJ, Christoph MJ, Winkler M, Neumark-Sztainer D. Life Events and Longitudinal Effects on Physical Activity: Adolescence to Adulthood. *Med Sci Sports Exerc* 2019; **51**(4): 663-70.

10. Rookus MA, Rokebrand P, Burema J, Deurenberg P. The effect of pregnancy on the body mass index 9 months postpartum in 49 women. *Int J Obes* 1987; **11**(6): 609-18.

11. Rosenberg L, Palmer JR, Wise LA, Horton NJ, Kumanyika SK, Adams-Campbell LL. A prospective study of the effect of childbearing on weight gain in African-American women. *Obes Res* 2003; **11**(12): 1526-35.

12. Sidebottom AC, Brown JE, Jacobs DR, Jr. Pregnancy-related changes in body fat. *Eur J Obstet Gynecol Reprod Biol* 2001; **94**(2): 216-23.

13. Smith DE, Lewis CE, Caveny JL, Perkins LL, Burke GL, Bild DE. Longitudinal changes in adiposity associated with pregnancy. The CARDIA Study. Coronary Artery Risk Development in Young Adults Study. *JAMA* 1994; **271**(22): 1747-51.

14. Smith KJ, McNaughton SA, Gall SL, Otahal P, Dwyer T, Venn AJ. Associations between Partnering and Parenting Transitions and Dietary Habits in Young Adults. *J Acad Nutr Diet* 2017; **117**(8): 1210-21.

15. South-Paul JE, Rajagopal KR, Tenholder MF. Exercise responses prior to pregnancy and in the postpartum state. *Med Sci Sports Exerc* 1992; **24**(4): 410-4.
